# Microfluidic ocular formulation of voriconazole designed for hospital production

**DOI:** 10.1007/s13346-026-02091-z

**Published:** 2026-04-08

**Authors:** Louise Stinat, Marie Bonnin, Jean-Christophe Gimel, Amandine Gastebois, Bienvenue Razafimandimby, Emilie Tireau, Nolwenn Lautram, David Dallerac, Guillaume Lefebvre, Sylvain Verron, Brice Calvignac, Sylvie Crauste-Manciet, Frederic Lagarce

**Affiliations:** 1 Université d’Angers, CHU Angers, INSERM, CNRS, MINT, SFR, ICAT, F-49000 Angers, France; 2 CHU Angers, Département Pharmacie, 4 Rue Larrey, 49933 Angers Cedex 9, France; 3 Université d’Angers, Synnanovect, SFR ICAT, F-49000 Angers, France; 4 Université d’Angers, CHU Angers, IRF, SFR ICAT, F-49000 Angers, France; 5 Université d’Angers, LARIS, SFR MathSTIC, F-49000 Angers, France

**Keywords:** Nanoemulsion, Voriconazole, Fungal keratitis, Micelle, Microfluidics

## Abstract

Fungal keratitis is a serious corneal infection with a limited therapeutic panel. Voriconazole, an azole antifungal, has been shown to be effective against many of the fungal strains involved in this type of infection. The absence of an ocular dedicated formulation on the market has led to the use of the sterile commercial cyclodextrin based formulation as a hospital preparation with some drawbacks. The aim of this study was to design a dedicated formulation for ocular delivery using oil-in-water nanoemulsion. A 0.4% voriconazole nanoemulsion was formulated with an oil and two surfactants, complying with the standards set by the authorities and literature. The nanoemulsion was produced using the phase inversion composition method. It was also formulated using microfluidic technology, suitable for hospital transposition. The mean droplets size was around 100 nm and zeta potential was negative. The pH and osmolality have been adjusted without destabilizing the nanoemulsion. Additionally, it was demonstrated that the formulation consists of two compartments: oil nanodroplets and residual micelles. Voriconazole was preferentially found in the micellar compartment. Finally, in vitro determination of minimal-inhibition-concentration tests were carried out on *Aspergillus* and *Fusarium* fungal species and compared to the 1% current hospital preparation. These tests showed that our formulation was no less effective than the hospital preparation.

**Graphical abstract:**

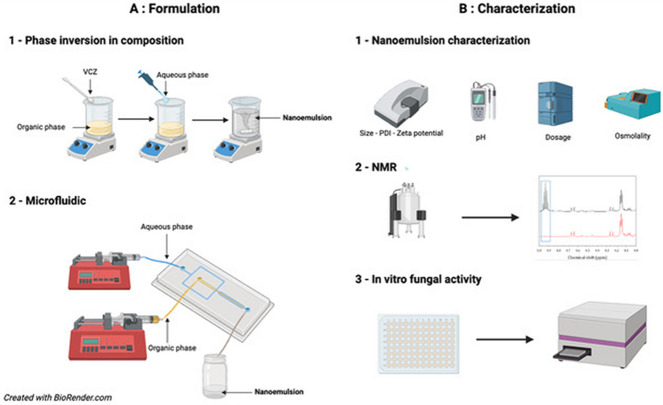

**Supplementary Information:**

The online version contains supplementary material available at 10.1007/s13346-026-02091-z.

## Introduction

Fungal keratitis is a serious cornea infection caused by yeasts or filamentous fungi. It can lead to blindness in affected patients. The strains most commonly encountered in this type of infection are ubiquitous filamentous environmental fungi (*Fusarium spp* and *Aspergillu*s *spp)* that invade the cornea epithelium and then can penetrate into the stroma [1]. In developed countries, contact-lens wear is the main cause of fungal keratitis, and the risk increases with prolonged lens contact or poor hygiene. Extended corticosteroid treatment can also increase the risk of developing fungal keratitis [2]. The treatment of fungal keratitis is long and complicated. It is adapted to the severity of the lesion and the infectious species involved. The preferred active substances are Natamycin and Voriconazole (VCZ). However, as the therapeutic arsenal is very limited, the eye drops available to treat fungal keratitis must be prepared by hospital pharmacies [1, 3, 4].

VCZ is a molecule of choice in the treatment of fungal keratitis, thanks to its broad spectrum of action [2, 4]. It is an antifungal compound of the triazole family, acting on the fungal membrane to inhibit demethylation of 14 alpha-lanosterol, thus inhibiting biosynthesis of fungal ergosterol, an essential component of the fungal membrane. VCZ can be applied topically, orally, or injected in case of severe infection. Currently, the only VCZ preparation for topical application is a 1% hospital preparation made from the VFEND^®^ commercial powder used for injection [4–6].

Topical treatment with VCZ requires very frequent applications, approximately 1 drop per hour. This dosage is due to the eye's physiology and its multiple physiological barriers that protect it from external aggression. Low ocular permeability and lacrimal clearance considerably reduce drug retention time in the eye, with instantaneous elimination of around 85% of the freshly administered active ingredient [7, 8]. However, this repeated administration of eye drops can reduce patient compliance, leading to treatment failure.

For several years now, innovative formulations, notably nanoformulations such as nanoemulsions and nanoparticles, have been under development to increase the residence time of active ingredients in the eye, to improve management and to facilitate patient compliance [8, 9]. Nanoemulsions are among the innovative formulations that offer several advantages. Nanoemulsions are a mixture of two immiscible liquids, resulting in the dispersion of one of them in the form of nanodroplets in the other, stabilized by a surfactant. There are two types of nanoemulsion: water-in-oil (W/O) or oil-in-water (O/W). O/W nanoemulsions can enhance the bioavailability of lipophilic, poorly water-soluble active ingredients such as VCZ. The active ingredients are first solubilized in oil, then dispersed in an aqueous phase to form the final O/W nanoemulsion [6]. The use of nanoemulsions has been shown to improve ocular drug bioavailability, and to increase drug residence time on the ocular surface. Both features are sought in the targeted clinical application, the long term objective of our project is to deliver the drug at corneal surface in order to have a prolonged action on the epithelium and to have an enhanced permeation into the stroma, if the infection is to deep voriconazole is however administered via intrastromal injection, this point will be discussed at the end of the present paper [6, 10, 11].

There are various methods for producing nanoemulsions, which can be divided into 2 categories. The first one includes the high-energy techniques such as ultrasonication and high-pressure homogenization [12]. These methods require expensive equipment and substantial energy input. They are better suited to industrial-scale production. In contrast, in a hospital pharmacy environment, low-energy methods such as spontaneous emulsification may be a more accessible technology for producing nanoemulsions [12]. Additionally, the phase inversion composition (PIC) method, which is typically performed as a batch process [13], can be easily adapted for continuous processing using microfluidics [14]. Microfluidics refer to systems that use small volumes of liquid—from a few microliters to a few milliliters—through microchannels. This technology allows for the production of standardized nanoemulsions by precisely controlling the hydrodynamic and thermodynamic parameters of the involved phases. In addition, microfluidics is a process that can be easily optimized, in particular by adjusting the size of the droplets formed, thanks to the existence of different channel structures [15]. The advantages of microfluidics are: an economical system thanks to the production method and the low volume of excipients used, and “automation” of the process limiting human intervention and errors [16], making it the method of choice for hospital production.

The aim of this preliminary formulation work was twofold: first, to produce a VCZ nanoemulsion using a low-energy PIC process; and second, to produce this nanoemulsion using microfluidics with the goal of transposing it to a hospital pharmacy. The combination of the low-energy PIC process and recent developments in 3D printing has made microfluidic chips cost-effective. Furthermore, microfluidics enable highly reproducible preparation at the patient's bedside. Finally, the aim is to determine the minimum inhibitory concentrations (MICs) on fungal strains in order to compare the performance of the hospital preparation and the hereby proposed nanoemulsion of VCZ.

## Materials

VCZ was purchased from Fischer Scientific SAS (United States). Labrasol^®^ was gifted by Gattefosse (France). Labrafac^®^ WL1349 was purchased from Gattefosse (France). Polysorbate 80, Isopropyl Myristate, Glycerol, Cremophor^®^ EL35 and deuterated chloroform were purchased from Sigma-Aldrich (Germany). Captex^®^ 8000 NF was gifted by Abitec Corporation (United State). All these products were used as received. RPMI 1640 medium, MOPS, L-Glutamine was purchased from Sigma (Germany), DMSO and VCZ from Thermo-Fischer (France). VCZ was prepared in DMSO to obtain a solution at 2 mg/mL. VCZ powder for injection from Stragen (France) was reconstituted with 19 mL MilliQ^®^ water to obtain a 1% formulation in line with hospital preparation. A 0.006% sodium chloride (NaCl) solution was obtained by diluting NaCl 0.9% from Fresinus (France) with Milli-Q^®^ water. O NaOH purchased from Fresenius (France), in Mili-Q^®^ water. Milli-Q^®^ water was obtained by Milli-Q^®^ Advantage A10 water purification system from Merck-Millipore (United-States). The microfluidic chip used in the formulations was produced at the laboratory by 3D printing (see Method section for details).

## Methods

### Formulation and characterization of nanoemulsions

#### Solubility tests

Preliminary solubility tests were carried out to select the most suitable excipients for VCZ solubilization. A spatula tip of active substance (approx. 2 mg) was added to 1 mL of each of the excipients tested. The mixture was then heated in a water bath at 50 °C for 1 h, alternating with vortex stirring to resuspend the active substance. For excipients that had solubilized their VCZ in less than one hour, increments of 5 mg of VCZ were then added. After one hour, the mixtures were visually examined to check for any visible, insoluble particles of VCZ. Solubilities were determined for selected excipients after screening to select excipients capable of PIC process and which were not toxic to the eye.

#### Preparation of organic and aqueous phases

The organic phase of nanoemulsion contains an oil, a surfactant and optionally a co-surfactant. To prepare the organic phase, the oil, surfactant and co-surfactant are weighed together. The organic phase is heated to 50 °C and stirred with a magnetic stirrer at 500 rpm. If VCZ is added, it is weighed into the organic phase and solubilized with a magnetic stirrer at 500 rpm for 45 min to 1 h until completely solubilized. The aqueous phase used contains Mili-Q^®^ water and, when osmolality adjustment is required, glycerol. To obtain osmolality in these formulations containing 80% water, 2.25% (w/v) glycerol was weighed and diluted in Mili-Q^®^ water prior to the PIC formulation process (described in Section "Preparation nanoemulsions using batch PIC process").

#### Size measurement, polydispersity index and zeta potential analysis

Zeta potential, size and polydispersity index (PDI) were measured with the Zetasizer Ultra Red (Malvern Instrument, Germany). Samples were analyzed at 25 °C, with a 173° scattering angle, by Dynamic Light Scattering after appropriate dilution of samples in Mili-Q^®^ water to suppress multiple scattering. Z-average diameter, *i.e.* the intensity-weighted mean hydrodynamic size from 3 consecutive measurements and associated PDI, were obtained from the cumulant analysis. The zeta potential was obtained from electrophoretic light scattering (ELS) measurement after diluting samples in a 0.006% NaCl mixture to achieve sufficient conductivity (> 0.1 mS/cm).

#### Osmolality and pH measurements and adjustment

Osmolality is measured with the VAPRO^®^ 5520 vapor pressure osmometer from Wescor (United States). The pH of the nanoemulsion is measured with the SevenDirect SD20 pH meter from Mettler-Toledo (France). All measurements were performed after instruments calibration. Nanoemulsion pH was adjusted by adding drop by drop a 1 M NaOH solution to obtain a pH close to 7. Iso-osmolality is obtained by adding 2.25% (w/v) glycerol.

#### Purification by tangential flow filtration

Tangential flow filtration (TFF) was carried out using the Äkta flux system from GE Healthcare Bio Sciences A (Sweden). It is used to purify samples and separate nanoemulsions (retentate) from micelles and other particles smaller than the 750 kDa filtration membrane pores (permeate).

A 10 mL sample of nanoemulsion containing 40 mg of VCZ is added to a container and topped up to 200 mL with Milli-Q^®^ water to obtain a 1:20 dilution and reduce the filtration membrane clogging. TFF is then carried out in two steps:

- Diafiltration step: a Mili-Q^®^ water supply is programmed to maintain a constant volume of 200 mL of the sample. This operation takes 1 h at a flowrate of 100 mL/min in the circulating line and a transmembrane pressure of 0.6 bar. Small particles and micelles pass through the 750 kDa membrane pores and are removed in the permeate. Oil nanodroplets are retained. Around 300 mL were recovered by diafiltration.
- Concentration step: during this step, the water supply is stopped. The sample follows the same circuit. When 190 mL of permeate have passed through the membrane, the 10 mL of starting sample is recovered. This corresponds to the retentate containing the purified nanoemulsions.

#### Determination of the micelle loading ratio

The micelle loading ratio was calculated using the following equation:

$$\frac{{m(VCZ)}_{\mathrm{permeate}}}{{m(VCZ)}_{\mathrm{initial}}}\times 100=\frac{VCZ\, permeate\;dosage\, \left(mg\right)}{VCZ\, initial\;concentration\;\left(mg/ml\right)\times 10\, \left(mL\right)}\times 100$$

With m(VCZ)_permeate_ the mass of VCZ recovered in the permeate from the UPLC dosage and m(VCZ)_initial_ the introduced mass of VCZ in the formulation before filtration.

The dosage of VCZ is obtained by UPLC assay.

#### Nuclear magnetic resonance (NMR)

Permeates (around 500 mL) were first evaporated using a Stuart sample concentrator SBHCONC/1 (Barloworld Scientific, Sandton, South Africa), then freeze-dried using a Lyovac GT2 freeze dryer (Steris, USA), and finally redissolved in CDCl3 for ^1^H NMR analysis. Spectra were acquired on a 500 MHz Avance III HD NMR spectrometer (Bruker, France) equipped with a 5 mm Observe broadband probe. ^1^H NMR spectra of all ingredients and VCZ were also acquired at 1% w/v for comparison.

#### UPLC analysis

Samples were analyzed by ultra-performance liquid chromatography (UPLC) on Acquity UPLC^®^ H-Class Bio system from Waters^®^ (United States). Separation and quantification were carried out using the Acquity UPLC^®^ BEH C18 reverse phase (1.7 μm × 2.1 × 50 mm) from Waters^®^ (United States). The method uses an isocratic gradient at 40 °C. The analysis wavelength was 256 nm. The mobile phase was composed of 0.1% acetic acid and acetonitrile in a 60/40 (v/v) ratio, with a flow rate of 0.3 mL/min and injection volume of 2 μL. The retention time of VCZ was obtained at 1.4 min. The matrix effect of nanoemulsion was studied preliminary and was checked by superimposing the calibration curves of free VCZ and VCZ with nanoemulsion (Figure S1 supplementary data). The UPLC method was validated. Linearity, precision and accuracy were determined. Linearity was demonstrated by five calibration solutions of 20, 40, 60, 80, 100 µg/mL. Linearity was assessed by the correlation coefficient (R^2^) and R^2^ > 0.999 on the five calibrations. Linear regression was calculated using data analysis software. Intra-day repeatability was determined using five calibration solutions per day. Inter-day repeatability was determined by repeating the experiments on three days. Inter-operator variability was assessed by repeating the tests with a second operator. The limit of detection (LOD) and the limit of quantification (LOQ) were determined from the background noise as the concentration as which the signal–noise ratio was 3:1 and 10:1 respectively. The LOD and the LOQ were 3.51 and 11.53 ng/mL respectively.

### Production processes and sterilization

#### Preparation nanoemulsions using batch PIC process

Nanoemulsions obtained by the batch PIC process [13] are called batch formulations in the following paragraphs. The organic phase (oil, surfactant, co-surfactant) was heated at 50 °C to reduce its viscosity. Once the desired temperature was reached, the organic phase was homogenized by mechanical agitation using a magnetic bar at 500 rpm. Finally, the aqueous phase, at room temperature, was added all at once and stirring was increased to 1100 rpm for one minute, then decreased to 600 rpm for one minute.”

#### Preparation of nanoemulsions using microfluidic PIC process

The microfluidic process was investigated in comparison to the batch PIC process to produce nanoemulsions. Compared to the batch process, nanoemulsions are still obtained by the PIC method but the organic and aqueous phases are mixed through a microchannel instead of a mechanical stirring.

**Fig. 1:**
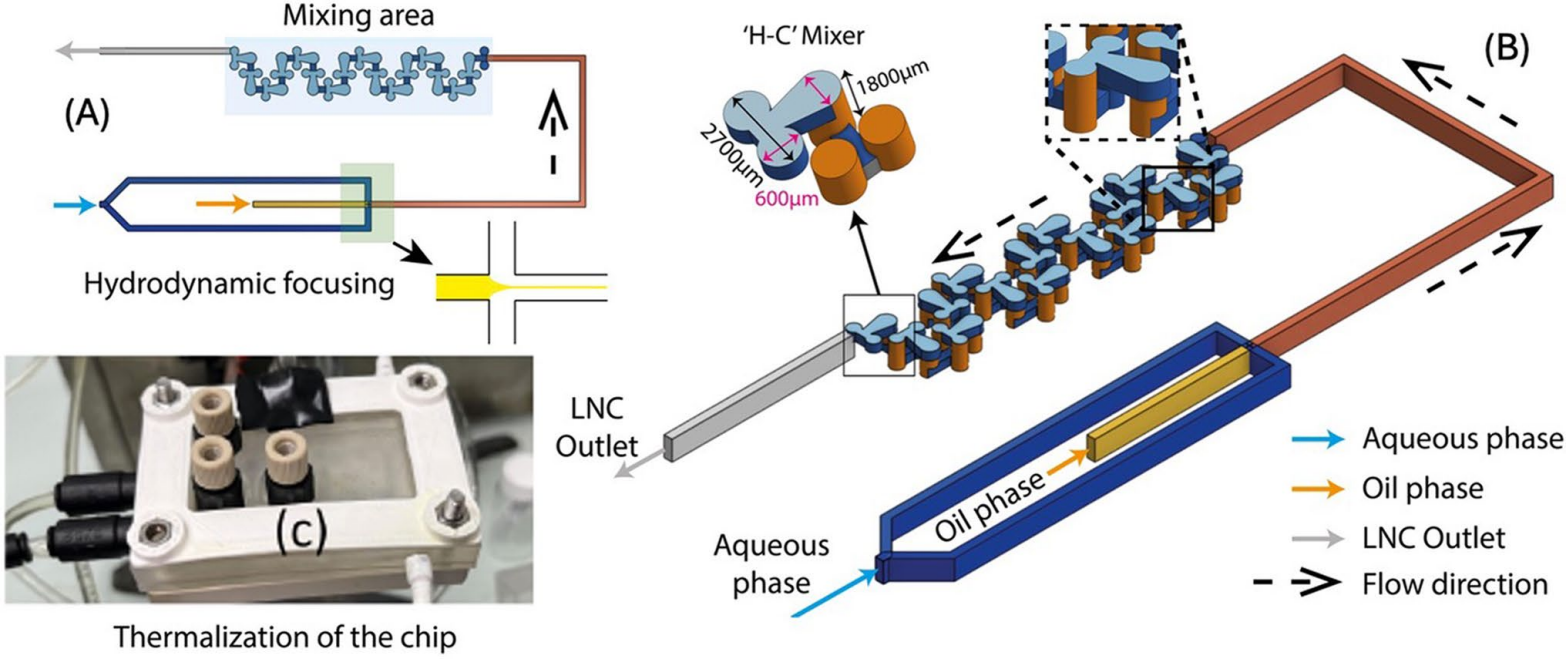
Microfluidic flow focusing chip: **A** 2D view ; **B** 3D view; **C** picture of the chip within its thermalization system

The organic phase is injected at 1 mL/min using the PHD 2000 infusion syringe pump from Harvard Apparatus (United States). The aqueous phase is injected using the PHD ultra syringe pump from Harvard Apparatus (United States). The flow rate of the aqueous phase is obtained by considering the density and flow rate of the organic phase and is set to 5.613 mL/min to have the same oil–water ration than with batch process. Syringes are maintained at 50 °C using a water circuit heated by the Haake DL 30 water bath from Thermo Haake (Germany). The microfluidic chip and all other fluidic and thermalization components were 3D printed and are presented in Fig. 1.

Firstly, chips were designed by CAD (Computer Aided Design) using Onshape software (Onshape, USA) and then modelled for printing during slicing using PreForm software (Formlabs, USA). The Formlabs 3B + SLA printer was used to manufacture these devices in transparent UV-curable polyacrylic resin (Formlabs CLEAR, part number RS-F2-GPCL-04, USA). The devices were then post-treated by rinsing with isopropyl alcohol in a tank (Formlabs Form Wash, USA) and by a final photopolymerization by UV treatment in a chamber (Formlabs Form Cure, USA). The design adopted for the microfluidic chip channels is the ‘H-C’ type, for which some of new technical and geometry modifications were brought in comparison with the crenelated microfluidic chip developed previously in the lab [14]. Indeed, following the proof-of-concept of the feasibility of formulating nanoemulsions in microfluidics printed chips, new developments have been made to improve the printing resolution of the micromixing device and to drop the chip production costs to few euros in mind the translational objective to produce nanomedicines. The design evolution of the micromixer device consists in *(i)* switching to 3D chip printing using stereolithography (SLA), for a better prototyping resolution of the chip and respectful of the design by CAD; *(ii)* increasing the aspect ratio of channels sections to 600 μm width and 1800 μm in depth; *(iii)* replacing the 45 cm long 2D crenelated channels path previously used by a 6 cm path including passive 3D H-C micromixers that split and recombine the flow (Fig. 1) with a mixing efficiency above 90% at low Reynolds numbers [17]; *(iv)* replacing the Y fluidic contact junction between the oil and the aqueous phase by a hydrodynamic flow focusing inlet that reduces the molecular diffusion path-length and mixing times [18, 19]. The CAD files are provided as supplementary data.

#### Preparation of micellar solution

Micellar solution is formulated with only surfactants that were added in the same quantities as in the nanoemulsion. The oil was replaced by additional water. The VCZ was added in the surfactant to be solubilized. The preparation process was similar to the batch process.

#### Sterilizing filtration

Sterilization tests were carried out on the final formulation with sterilizing filtration of the nanoemulsion using a 0.22 µm pore filter.

#### Viscosity

Viscosity measurements were performed at 25 °C using a Kinexus rotational rheometer (Netzsch, Prime Pro + model) equipped with a cone–plate geometry (2° angle, 60 mm diameter). Steady-state flow curves were obtained from 11 measurement points over a shear rate range of 10 to 100 s⁻^1^. An equilibration time was applied at each shear rate to ensure that steady-state conditions were reached.

### In vitro fungal activity

The minimum inhibitory concentration (MIC) studies were carried out on 4 different fungal species: *Fusarium solanii* (IHEM 15591), *Fusarium oxysporum* (IHEM 17953), *Aspergillus flavus* (155,354,818–09)*, Aspergillus fumigatus* (116,191,047–01). All the fungal species were isolated from the human ENT area.

Briefly, fungal species were cultivated in Potatoe dextrose agar medium (Condalab) during 3 days at 37 °C for *Aspergillus* species and during 5 days at 25 °C for *Fusarium* species. Fungal spore suspensions were prepared by scratching plates in 10 mL of sterile water to obtain an OD between 0.06 and 0.1 at 630 nm and filtered on miracloth (22 µM) to remove hyphae. Spores were then diluted 1:10 in a sterile 1X RPMI 1640 solution medium supplemented with 2 mM L-Glutamine, buffered with MOPS.

The Clinical and Laboratory Standard Institute (CLSI) protocol was used to determine MICs. Several controls were used: a growth control, a sterility control, a positive control containing VCZ at 2 mg/mL prepared in a DMSO solution, the 1% VCZ hospital preparation and blank nanoemulsions. The solutions tested were 0.4% nanoemulsions produced in batch and in microfluidic. MICs were determined at 630 nm in flat-bottomed 96-well plates. In the first well, 195 µL of sterile RPMI 1X and 5 µL of one of the tested solutions were plated. In wells 2 to 12, 100 µL of sterile RPMI was added. A dilution cascade was carried out from the first well by taking 100 µL from well number 1 into well number 2, etc. Finally, 100 µL of RPMI seeded with spores was added to each well. This procedure was carried out for each solution (controls and nanoemulsions) and each fungal species. For each experiment, the wells containing the nanoemulsions, the VCZ hospital preparation and blank nanoemulsion were prepared in technical triplicate and three independent experiments were performed (biological replicates). The plates were incubated in a wet chamber for 72 h Thermo Scientific incubator (Germany) at 25 °C for *Fusarium solannii and Fusarium oxysporum* and 37 °C for *Aspergillus flavus and Aspergillus fumigatus*.

MICs were determined after incubation for 72 h as the lowest concentration of sample with voriconazole to result in a growth reduction of 50% relative to growth control in antifungal-free RPMI. Growth was assessed by measuring the OD at 630 nm after 5 s of shaking with a Thermo scientific multiskan GO plate reader.

### Statistical analysis

All experiments were performed in triplicate at least three times independently. Data is expressed as the median ± standard deviation. Statistical analysis was performed using Jasp software (JASP 0.19.1, University of Amsterdam, Netherlands, 2024). A one-way analysis of variance and a Dunn's post-hoc comparison based on individual Mann–Whitney tests were used to evaluate differences between strains. In all analyses, p-values ≤ 0.05 were considered statistically significant.

## Results

### Selection of formulation excipients and VCZ final concentrations

The oils and surfactants were selected according to 2 criteria: their capacity to solubilize the VCZ [20] and their spontaneous emulsification properties [21]. The preliminary solubility tests for the oil were carried out with Isopropyl myristate, Labrafac^®^ WL1349 and Captex^®^ 8000. The tested surfactants were Labrasol^®^, Cremophor^®^ EL 35 and Polysorbate 80. Solubility results are summarized in Table 1.

**Table 1:** Estimated solubility of VCZ in the selected excipients

Excipient	Oils			Surfactants		
	Labrafac^®^ WL1349	Captex^®^ 8000	Isopropyl Myristate	Crémophor^®^ EL35	Polysorbate 80	Labrasol^®^
Estimated solubility	< 10 mg/mL	≈ 10 mg/mL	≈ 10 mg/mL	≈ 15 mg/mL	≈ 15 mg/mL	≈ 26 mg/mL

Based on these preliminary results, isopropyl myristate, Captex^®^ 8000 and the 3 surfactants were selected for nanoemulsion formulation. The organic phase was prepared by mixing one oil with one surfactant or one oil with two surfactants. All combinations were tested and the quantities of excipients were chosen in accordance with the competent authorities requirements and with data from the literature [10, 22, 23], in order to respect ocular tolerance. Stability was observed macroscopically at the time of preparation (emulsification or absence of emulsification) and observation of a creaming phenomenon after formulation. Formulations were considered stable when they were not destabilized after 24 h. Stability results are shown in Table 2.

**Table 2:** Stability of excipients in Captex^®^ 8000 and Isopropyl Myristate

	Captex^®^ 8000	Isopropyl Myristate
*Crémophor*^*®*^ *EL35*	No emulsification	No emulsification
*Polysorbate 80*	No emulsification	No emulsification
*Labrasol*^*®*^	No emulsification	No emulsification
*Crémophor*^*®*^ *EL35* + *Polysorbate 80*	Stable	Stable
*Crémophor*^*®*^ *EL35* + *Labrasol*^*®*^	Stable	Stable
*Polysorbate 80* + *Labrasol*^*®*^	Creaming after 24 h	Stable

Following those stability studies, it was decided to use isopropyl myristate rather than Captex^®^ 8000, as the latter is slightly irritating to the eyes [24]. The formula containing Labrasol^®^ and polysorbate 80 was chosen because Labrasol^®^ had a better solubility profile than Cremophor^®^ EL35 (Table 1).

Maximum allowed quantities for each excipient that were compatible with the toxicity standards [10, 22, 23] have been used to promote the solubility of VCZ and to allow the maximum drug loading. Thus, the final nanoemulsion was composed of 5%(w/v) isopropyl myristate, 4%(w/v) polysorbate 80 and 3%(w/v) Labrasol^®^. These proportions will be used for all future formulations.

Following the solubility tests, 5 mg/mL VCZ were solubilized in the organic phase. After formulation, VCZ precipitates were observed in the vial/tube less than 24 h after preparation. A VCZ concentration of 4 mg/mL was then tested. No precipitate was observed after 24 h or on the following days. It was therefore decided to formulate nanoemulsions at 4 mg/mL VCZ loading, *i.e.* 0.4% (w/v) for the remainder of the experiments.

### Nanoemulsion characterization

#### Batch process

The nanoemulsions were formulated without VCZ (blank nanoemulsions) or with VCZ. They were then characterized in terms of size, PDI, zeta potential, pH, osmolality and VCZ concentration (Table 3). Additional tests were carried out on the blank nanoemulsion by adjusting the pH and osmolality. Based on these results, it was decided to adjust the VCZ-loaded nanoemulsions directly.

**Table 3:** Physicochemical properties of nanoemulsions produced in batch (Mean ± SD, n = 3)

	Size (nm)	PDI	Zeta potential (mV)	pH	Osmolality (mmol/kg)	VCZ concentration (mg/mL)
Blank	101 ± 11	0.18 ± 0.02	- 14.2 ± 0.8	3.36 ± 0.04	145 ± 3	-
Adjusted blank	99 ± 12	0.16 ± 0.02	- 25.1 ± 0.6	7.6 ± 0.2	352 ± 22	-
With VCZ	104 ± 4	0.13 ± 0.02	- 25.58 ± 2.52	7.2 ± 0.2	370 ± 5	4.30 ± 0.08

After formulation, nanoemulsions were visually homogeneous. For the blank and the VCZ-loaded nanoemulsions, the droplet size was around 100 nm with a PDI of 0.175 and 0.134, respectively (Table 3). The zeta potential of the blank nanoemulsion was negative. The pH of the blank nanoemulsion before any adjustment was acidic and this nanoemulsion was hypo-osmolar. After adjustment of the VCZ- loaded nanoemulsion, a neutral pH of 7 and a slight hyper-osmolality was obtained, within the tolerance threshold for the eye [25]. The zeta potential became more negative after adjusting the pH (Table 3). Finally, the dosage showed that the formulation with VCZ loaded is 4.3 mg/mL, i.e. 0.43% (w/v), close to the theoretical concentration of 4 mg/mL.

#### Microfluidic process

Both formulations prepared by microfluidic process, with or without VCZ, have a comparable droplet size and PDI (Table 4). The same order of magnitude of zeta potential, pH and osmolality as for batch production was found. After adjustment, a neutral and slightly hyper-osmolar VCZ-loaded nanoemulsion at 0.431% (w/v) was obtained.

**Table 4:** Physicochemical properties of nanoemulsions produced by microfluidics (Mean ± SD, *n* = 3)

	Size (nm)	PDI	Zeta (mV)	pH	Osmolality (mmol/kg)	VCZ concentration (mg/mL)
Blank	91 ± 8	0.14 ± 0.06	- 18.21 ± 0.80	3.5 ± 0.04	143 ± 3	-
With VCZ	84 ± 7	0.12 ± 0.04	- 24.26 ± 2.46	7.0 ± 0.10	369 ± 20	4.31 ± 0.26

#### Effect of sterilization by filtration on nanoemulsions

The sizes measured post filtration on 0.22 µm pore filter is comparable to the sizes obtained before filtration. VCZ concentrations approached 4 mg/mL, with 4.22 and 4.36 mg/mL for batch and microfluidic formulations (Table 5).

**Table 5:** Physicochemical properties of nanoemulsions with VCZ after sterilizing filtration produced on batch and microfluidic (Mean ± SD, *n* = 3)

	Size (nm)	PDI	Zeta (mV)	VCZ concentration (mg/mL)
Batch	106 ± 6	0.111 ± 0.007	- 25.56 ± 3.37	4.22 ± 0.14
Microfluidic	87 ± 8	0.126 ± 0.003	- 22.46 ± 2.49	4.36 ± 0.13

### Purification of nanoemulsions by TFF

#### Separation of different particles and quantification of VCZ

VCZ quantification in the retentate was found below the UPLC calibration range (< 20 μg/mL), which is negligible in view of the permeate quantification results. In fact, more than 80% of the VCZ is recovered into the permeate and is therefore not encapsulated in the nanoemulsions (Table 5). Moreover, in the permeate, size measurements indicated the presence of micelles ranging from 12 to 15 nm in size, with PDIs around 0.2, irrespective of the formulation process (Table 6).

**Table 6:** Physicochemical characteristics and distribution of VCZ after filtered nanoemulsion purification (Mean ± SD, *n* = 3)

	Retentate		Permeate		
	Size (nm)	PDI	Size (nm)	PDI	Dosage of VCZ (mg)
Batch	120 ± 6	0.13 ± 0.02	16 ± 2	0.21 ± 0.02	34.20 ± 4.36
Microfluidic	113 ± 26	0.10 ± 0.03	14 ± 3	0.22 ± 0.18	38.56 ± 5.24

After characterization, micelle loading ratio could be calculated for each formulation. All micelle loading ratio were > 80% (Table 7).

**Table 7:** VCZ micelle loading ratio in microfluidic and batch processes on filtered nanoemulsion (Mean ± SD, *n* = 3)

	Micelle loading ratio (%)
Batch	80.96 ± 8.96
Microfluidic	88.43 ± 11.54

The same experience was carried out on unfiltered VCZ-loaded nanoemulsions to verify the impact of the sterilizing process using filtration. The same results were found (Table S1 and S2 in Supplementary Information), demonstrating that filtration had no effect on micelle loading ratio.

#### Chemical characterization of the permeate by NMR

NMR analysis was performed on the permeates to better characterize the chemical nature of the micelles. The ^1^H spectra revealed the presence of VCZ, Labrasol^®^ and polysorbate 80 on the permeate. The oil, Isopropyl myristate, which was the main component of the organic phase, was found in trace amounts. The other ingredients were present in the same proportions as in the organic phase (see Figures S2 and S3 in Supplementary Information). Permeates analysis were performed on all nanoformulations (batch and microfluidics) and the same results were found.

#### Formulation of a micellar solution for further investigations on the results

Because the concentration of VCZ in the oil nanodroplet compartment was very low and the oil (isopropyl myristate) was virtually absent from the VCZ-loaded micelles, it was decided to try encapsulating VCZ directly in micelles without using oil. The obtained micelles measured 10 ± 0.1 nm with a PDI of 0.171 ± 0.014 *(Mean* ± *SD, n* = *2*). This confirms that the used surfactant concentrations were above the critical micellar concentration: 13 to 24 mg/mL and 42 mg/mL for polysorbate 80 and Labrasol^®^ respectively [26, 27].

After 24 h, VCZ precipitates were observed at the bottom of the vial of the 0.4% VCZ micellar suspension. Formulations at 0.3% and 0.2% were carried out and showed no VCZ precipitate within 24 h of formulation or in the following days.

With this micellar solution, we were unable to micellize 100% of VCZ without oil and this seems to confirm the results presented with only 80% of VCZ-loading micelles (Table 7). Furthermore, it is not possible to optimize the micellar solution by increasing the surfactants, as the maximum concentrations of surfactants that are not toxic to the eyes were already used.

#### Viscosity of the nanoformulations

Rheological measurements showed stable and reproducible mean shear viscosities for both preparation methods, with values of 1.73 ± 0.04 mPa·s for batch samples and 1.94 ± 0.04 mPa·s for microfluidic ones. The formulations exhibited a newtonian behavior over the entire investigated shear rate range. The slight increase in viscosity for the microfluidic formulations may reflect differences inherent to the hydrodynamic environment and mixing intensity during production, but remains within a narrow range, demonstrating that both approaches yield formulations with comparable rheological properties.

### In vitro antifungal activity of the nanoemulsions

In vitro efficacy of batch-formulated and microfluidic nanoemulsions were compared with the hospital formulation (Table 8).

**Table 8:** Minimum inhibitory concentration (MIC—µg/mL) determined by the broth microdilution method for each formulation (hospital preparation, batch and microfluidic nanoemulsion) and fungal species (Mean + SD, *n* = 9)

	Hospital preparation	Batch	Microfluidic
*Fusarium oxysporum*	1.07 ± 0.24	0.88 ± 0.16	0.89 ± 0.18
*Fusarium solanii*	3.52 ± 0.87	**2.32 ± 0.44** (*p-value 0.014)*	2.51 ± 0.59
*Aspergillus flavus*	0.27 ± 0.17	0.30 ± 0.45	0.34 ± 0.19
*Aspergillus fumigatus*	0.57 ± 0.15	0.52 ± 0.10	**0.38 ± 0.10** (*p-value 0.003)*

Values in bold are those for which a significant difference was found between hospital preparations and nanoemulsions.

There was a significant difference between the nanoemulsion formulated in batch and the hospital preparation with the *Fusarium solanii* strain. The MIC was significantly lower. The same significative difference was obtained for the nanoemulsion formulated in microfluidic as compared to the hospital preparation for the *Aspergillus fumigatus* strain. No significant difference was found between batch and microfluidic nanoemulsions.

## Discussion

The first challenge in formulating the nanoemulsion was to solubilize VCZ. Indeed, it is a very poorly water-soluble molecule (0.098 mg/mL [27]). To formulate VCZ-based preparations, cyclodextrins have been proposed [28–30] because, by forming complexes with VCZ, they enable its water solubilization [30]. Thus, aqueous 1% VCZ ocular hospital formulation can be obtained from commercial forms like VFEND^®^, normally devoted to intra-venous administration and containing cyclodextrins. Cyclodextrins can however impair VCZ fungicide action if the drug remains too long associated in the complex. We thus wanted to avoid cyclodextrins and produce a nanoemulsion to allow the free drug to be active and to increase the residence time on the ocular surface [6, 10, 11]. Indeed, many daily administrations are necessary due to lacrimal clearance which results in compliance issues for the patient. To achieve this, excipients had to: *(i)* enable spontaneous self-emulsification, so that the nanoemulsion could be produced using low-energy processes such as PIC or microfluidics processes; *(ii)* solubilize VCZ; and *(iii)* be non-toxic to the eye. Eye toxicity standards impose strict limitations on the types and quantities of excipients that can be used in the final formulation. The best compromise was chosen by formulating a formulation with isopropyl myristate, polysorbate 80 and Labrasol^®^, yielding a 0.4%(w/v) VCZ nanoemulsion/micelle system. In vitro studies were required to confirm the formulation efficacy. Toxicity studies on rabbits will have to be carried out to demonstrate the good tolerance of our formulation.

Fungal keratitis is currently managed via a single hospital-produced preparation of VCZ 1%, which is made by reconstituting the commercial VCZ injectable formula [1]. Here, we have succeeded in formulating a 4 mg/mL-VCZ- loaded nanoemulsion/micelle system. The pH and osmolality of this formulation can be adjusted within the eye's tolerance range, without altering its physico-chemical characteristics. The pH of our formulation is around 7, compared to 5.76 for the hospital preparation. Regarding osmolality, we obtained a formulation of around 370 mOsm/kg versus 515 mOsm/kg for the hospital preparation. Thus, VCZ nanoemulsion/micelle system has a better tolerance profile regarding the eye's thresholds of acceptability [25]. Besides, we demonstrated that we could formulate a 0.4% VCZ-loaded nanoemulsion/micelle system using the microfluidic process. It was important to control this process if we were to produce this formulation in a hospital pharmacy, given the advantages of this method. In addition, the polymeric resin used to fabricate the 3D-printed chips is fully compatible with in-place cleaning using organic solvents such as ethanol or DMF, which effectively remove oily residues. Moreover, the selected resin is also available in a biocompatible and sterilizable version, which reinforced our initial choice of material for a potential hospital translation. However, the reusability of the chips still needs to be validated in future studies. In the meantime, single-use chips for each production batch remain a viable and economically acceptable solution for hospital pharmacy settings. Finally, we demonstrated that sterilization by sterilizing filtration was possible without modifying the nanoemulsion size, PDI or VCZ concentration. Rather than moist heat autoclaving, sterilizing filtration was considered to leave open the possibility of gelling our formulation. The aim would be to achieve a temperature-sensitive in situ gelation, producing a gel on contact with the ocular surface—from 32 °C [31, 32].

TFF purification coupled with permeate NMR characterization revealed that VCZ was preferentially located in surfactant-based micelles (between 80 and 90%). This distribution was not altered by the formulation process (batch or microfluidic) and/or sterilizing filtration. Recent studies have already revealed the presence of residual micelles with a similar low-energy phase inversion process [33]. Research is still underway to understand the mechanisms involved. Nevertheless, the present result calls into question the necessity of including oil in some formulations. Micelles of a similar size can be formulated without the use of oil. However, we were unsuccessful to formulate micelles loaded with 0.4% VCZ without adding oil, as precipitates were observed quickly after formulation. It would therefore appear that the oil helps to solubilize the VCZ, allowing loading of the remaining 20% of VCZ not found in the micelles. At last, the formulation of a nanoemulsion/micelle system requires the study and performance of ex vivo and in vivo tests to investigate the corneal permeation of VCZ-loaded micelles, which are much smaller than nanoemulsion droplets. These experiments will allow a future optimization of our formulation, especially on the charge of the micelles that can be adjusted. Indeed, we produced in this preliminary formulation micelles with a negative charge. This is a good point to avoid a high lacrimal clearance after protein tears adsorption but can impede the diffusion through the cornea especially through ocular mucins. Thus in vivo data are needed to allow the adaptation of the formulation.

In vitro tests were carried out on the species most encountered in fungal keratitis [1]. The results showed that our formulations had lower MICs for 2 strains *(Aspergillus fumigatus* and *Fusarium solanii),* demonstrating greater efficacy than the hospital preparation whereas its VCZ concentration was lower. The other MICs values found for all fungal species are similar between the hospital preparation and the nanoemulsions/micelle system formulated in batch and microfluidic. Although no significant difference was shown, the 0.4% VCZ nanoemulsions/micelle system appears to be at least as effective on fungal strains as the hospital preparation which contained 1% VCZ. Furthermore, the literature shows that nanoemulsions increase the residence time in the eye [6, 10, 11]. This is an advantage in favor of the proposed nanoemulsions, since theoretically they have a longer time on the ocular surface, and thus increasing their antifungal activity compared with the hospital preparation. To demonstrate this, it would then be necessary to carry out tests using ex-vivo or in vivo models to compare the residence times of the preparations on the eye. Finally, as mentioned above, the possibility of gelation of the nanoemulsions was left open because gelation would increase the retention time of the nanoemulsion on the ocular surface, thus improving its efficacy [31, 32, 34].

Our study is preliminary and has several limitations. Some data could not be studied and will need to be collected in the future. The stability of the preparation and its storage conditions must be defined as the final optimized formulation is obtained. Ex vivo and in vivo studies will need to be conducted to ensure that our formulation is well tolerated, but also to measure its efficacy and compare it with the hospital preparation currently in use. Finally, the kinetics of VCZ release from nanoemulsions/micelle system compared to that in cyclodextrins (hospital preparation) will be an interesting point to study and discuss.

## Conclusion

A 0.4% w/v VCZ nanoemulsion/micelle system has been formulated while adhering to ocular toxicity standards. In addition, VCZ-loaded nanoemulsion/micelle system were formulated using 2 different processes, including microfluidics, leaving open the possibility of future hospital transposition. Finally, in vitro tests showed that this VCZ nanoemulsion/micelle system was at least as effective as the hospital preparation.

## Supplementary Information

Below is the link to the electronic supplementary material.

ESM 1 (DOCX 649 KB)

ESM 2 (DOCX 1.84 MB)

## Data Availability

Data are stored and saved to be available, lab notebook is used to trace experiments, purchase documents of materials are archived. Data related to this study are available on request from the authors.
