# Supplementary material for: Microfluidic ocular formulation of voriconazole designed for hospital production
Source: Drug Deliv Transl Res. 2026 Apr 8;16(7):2488–98. doi: 10.1007/s13346-026-02091-z (PMC13294310; doi:10.1007/s13346-026-02091-z)
Supplement: Supplementary file 1 — (DOCX 649 KB) [file 13346_2026_2091_MOESM1_ESM.docx]

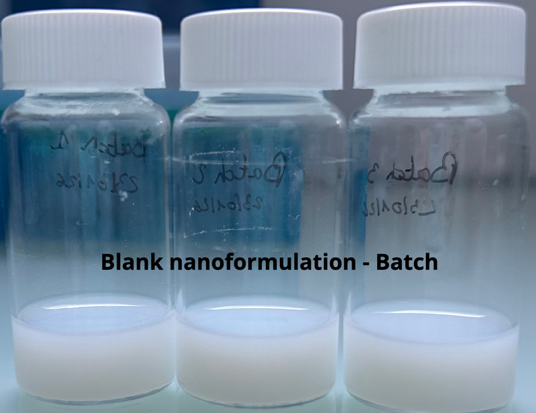


Supplementary picture A: Photograph of nanofomulation obtained after batch process


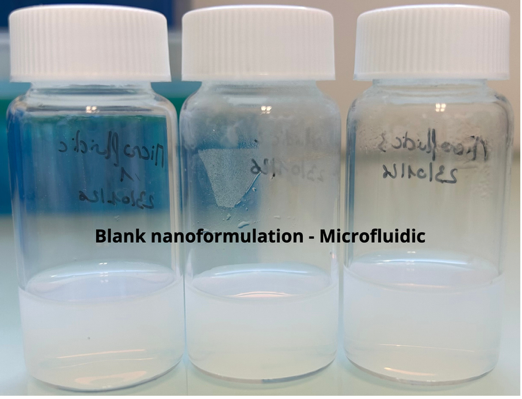


Supplementary picture B: Photograph of nanoformulation obtained after microfluidic process
