# Supplementary material for: Microfluidic ocular formulation of voriconazole designed for hospital production
Source: Drug Deliv Transl Res. 2026 Apr 8;16(7):2488–98. doi: 10.1007/s13346-026-02091-z (PMC13294310; doi:10.1007/s13346-026-02091-z)
Supplement: Supplementary file 2 — (DOCX 1.84 MB) [file 13346_2026_2091_MOESM2_ESM.docx]

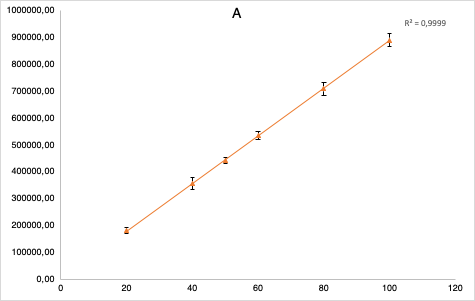
Supplementary information


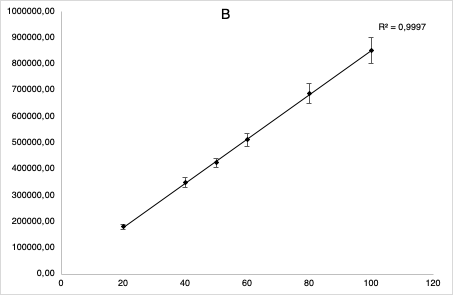


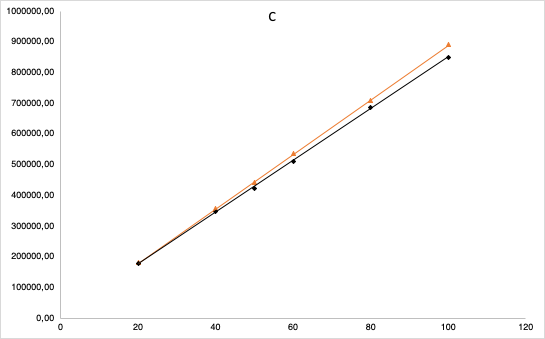


**Fig. S1** Calibration curves of VCZ with nanoemulsion and glycerol (A), VCZ (B) and superposition of both calibration curves to estimate matrix effect (C) (Mean ± SD, n=3).


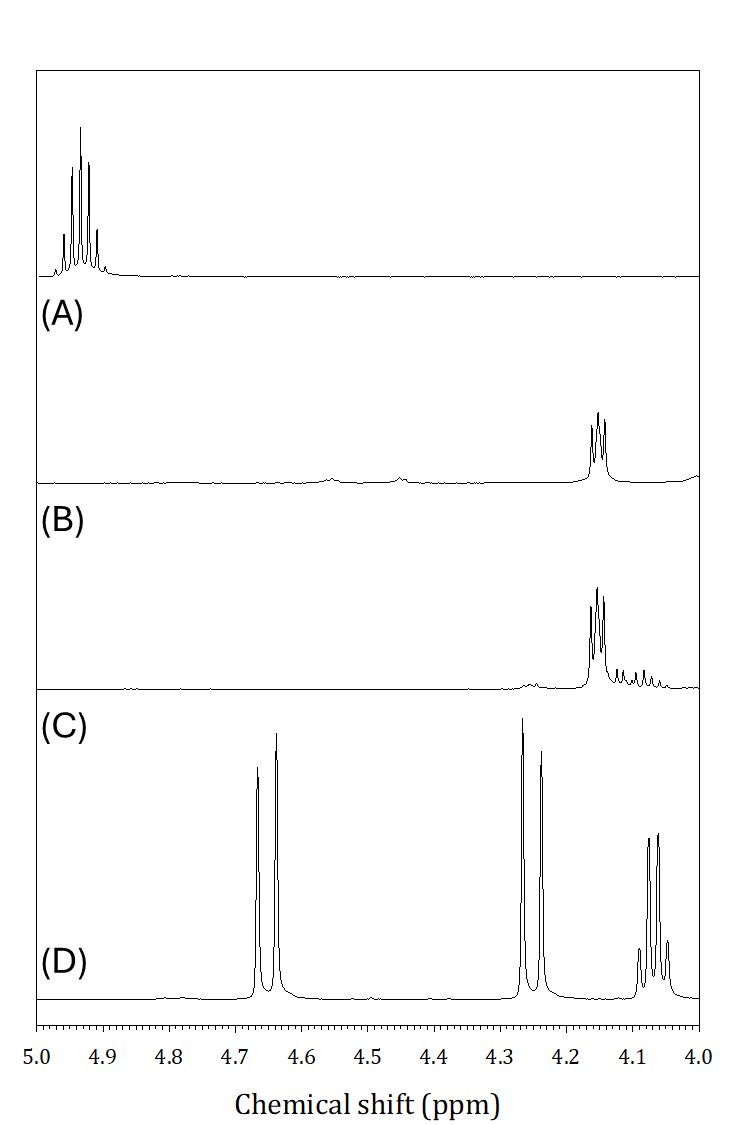


**Fig. S2** ^1^H NMR spectra of the various compounds in the organic phase. They are plotted in the relevant range from 5 to 4ppm where characteristic peaks are observed. All spectra were acquired from 1 % m/v solutions in CDCl_3_. (A) isopropyl myristate, (B) Tween80, (C) Labrasol, and (D) VCZ


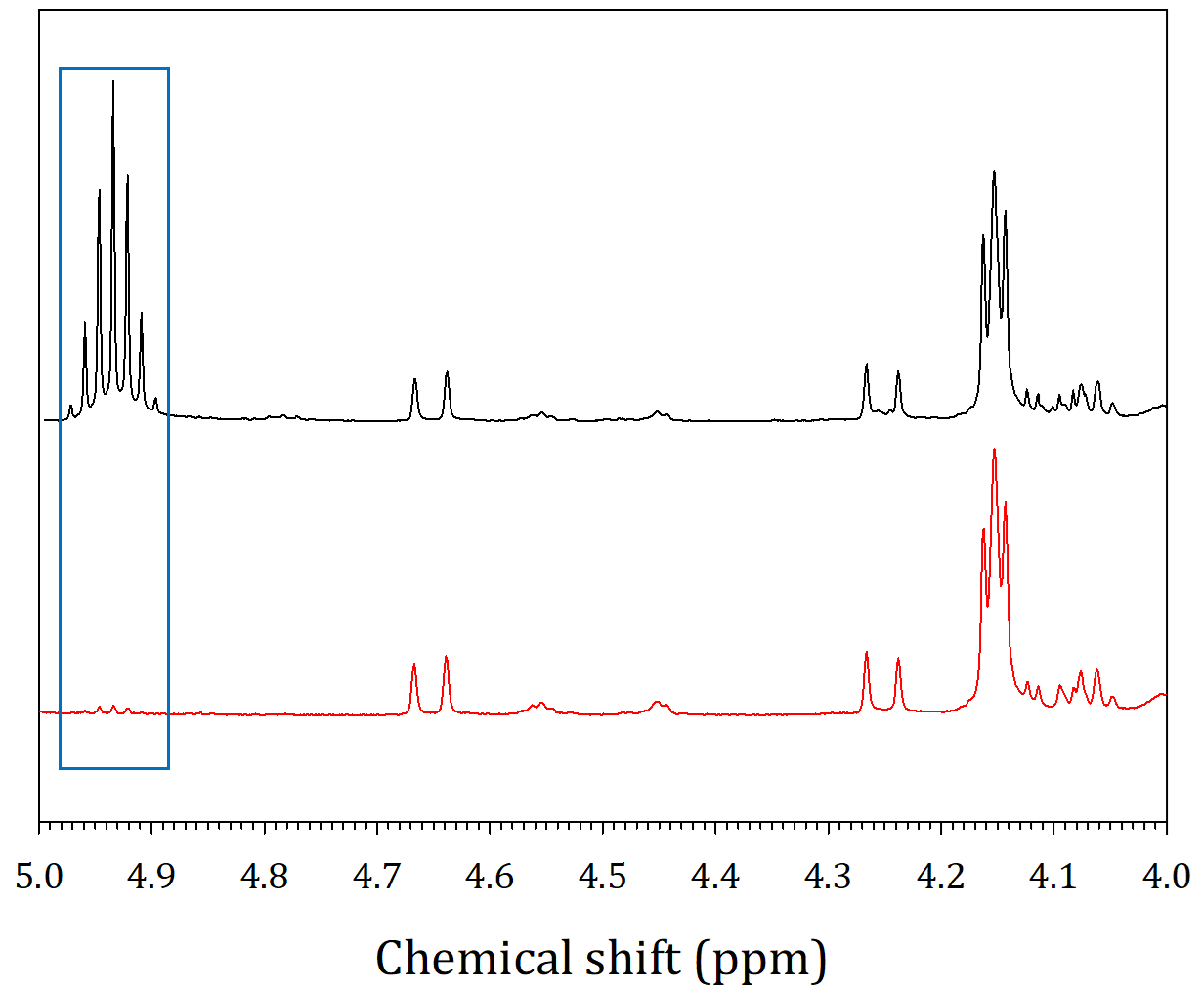

**Fig. S3** Comparison of ^1^H NMR spectra in CDCl_3_ for the organic phase (black) and the permeate (red) for the one sample permeate produced in batch and filtered by sterilizing filtration. The NMR spectrum of the organic phase was reconstructed from the spectra of each of the components (Fig. S2), weighted by their mass fraction in the organic phase. The blue frame indicates the resonance of a proton only present in isopropyl myristate.

**Table S1.** Physicochemical characteristics and distribution of VCZ after unfiltered nanoemulsion purification (Mean ± SD, n=3).

|  | Retentate |  |  | Permeate |  |
| --- | --- | --- | --- | --- | --- |
|  | Size (nm) | PDI | Size (nm) | PDI | Dosage of VCZ (mg) |
| Batch | 132 ± 13 | 0.097 ± 0.023 | 12 ± 1 | 0.174 ± 0.098 | 36.39 ± 5.83 |
| Microfluidic | 144 ± 7 | 0.065 ± 0.012 | 13 ± 1 | 0.172 ± 0.094 | 42.73 ± 2.45 |

**Table S2***.* VCZ micelle loading ratio in microfluidic and batch processes on unfiltered nanoemulsions (Mean ± SD, n=3)

|  | Micelle loading ratio (%) |
| --- | --- |
| Batch | 79.99 ± 8.01 |
| Microfluidic | 81.73 ± 0.11 |
